# Increased Oxidative Stress Underlies Abnormal Pain Threshold in a Normoglycemic Japanese Population

**DOI:** 10.3390/ijms21218306

**Published:** 2020-11-05

**Authors:** Sho Osonoi, Hiroki Mizukami, Chieko Itabashi, Kanichiro Wada, Kazuhiro Kudoh, Akiko Igawa, Saori Ogasawara, Yasuyuki Ishibashi, Makoto Daimon, Soroku Yagihashi, Shigeyuki Nakaji

**Affiliations:** 1 Department of Pathology and Molecular Medicine, Hirosaki University Graduate School of Medicine, 5 Zaifu-cho, Hirosaki, Aomori 036-8562, Japan; s.osonoi@hirosaki-u.ac.jp (S.O.); c-ita31@hirosaki-u.ac.jp (C.I.); spkkspkk@hirosaki-u.ac.jp (K.K.); saorioga@hirosaki-u.ac.jp (S.O.); yagihasi@hirosaki-u.ac.jp (S.Y.); 2 Department of Endocrinology and Metabolism, Hirosaki University Graduate School of Medicine, 5 Zaifu-cho, Hirosaki, Aomori 036-8562, Japan; mdaimon@hirosaki-u.ac.jp; 3 Department of Orthopaedic Surgery, Hirosaki University Graduate School of Medicine, 5 Zaifu-cho, Hirosaki, Aomori 036-8562, Japan; wadak39@hirosaki-u.ac.jp (K.W.); yasuyuki@hirosaki-u.ac.jp (Y.I.); 4 Department of Gastroenterological Surgery and Pediatric Surgery, Hirosaki University Graduate School of Medicine, 5 Zaifu-cho, Hirosaki, Aomori 036-8562, Japan; igawa-a@hirosaki-u.ac.jp; 5 Department of Social Medicine, Hirosaki University Graduate School of Medicine, 5 Zaifu-cho, Hirosaki, Aomori 036-8562, Japan; nakaji@hirosaki-u.ac.jp

**Keywords:** elevated pain threshold, diabetic polyneuropathy, oxidative stress, 8-hydroxy-2′-deoxyguanosine, normal-high HbA1c, inflammation

## Abstract

Normal-high HbA1c levels are a risk factor for attenuated pain sensation in normoglycemic subjects. It is unclear, however, what mechanisms underlie the pathogenesis of attenuated pain sensation in such a population. We, therefore, explored the relationship between oxidative stress (OS) and pain sensation in a rural Japanese population. A population-based study of 894 individuals (average age 53.8 ± 0.5 years) and 55 subjects with impaired fasting glucose (IFG) were enrolled in this study. Individuals with diabetes were excluded. Relationships between pain threshold induced by intraepidermal electrical stimulation (PINT) and clinico-hematological parameters associated with OS were evaluated. Univariate linear regression analyses revealed age, BMI, HbA1c, the OS biomarker urine 8-hydroxy-2′-deoxyguanosine (8-OHdG), systolic blood pressure, and decreased Achilles tendon reflex on the PINT scores. Adjustments for age, gender, and multiple clinical measures confirmed a positive correlation between PINT scores and urine 8-OHdG (β = 0.09, *p* < 0.01). Urine 8-OHdG correlated positively with higher HbA1c levels and age in the normoglycemic population. Unlike in the normoglycemic population, both inflammation and OS were correlated with elevated PINT scores in IFG subjects. OS may be a major contributing factor to elevated PINT scores in a healthy Japanese population.

## 1. Introduction

Overproduction of reactive oxygen species (ROS) in conjunction with the deterioration of antioxidant defenses causes an increased burden of oxidative stress (OS), leading to damages to membrane lipids, proteins, and DNA [1]. OS is directly linked to many physiological processes and pathological conditions, including diabetic polyneuropathy (DPN) [2,3,4]. In experimental DPN models, 8-hydroxy-2′-deoxyguanosine (8-OHdG) level, a biomarker to assess DNA damage induced by OS, was significantly increased in Schwann cells and endothelial cells of the sciatic nerve in diabetic animals relative to their nondiabetic counterpart [5,6,7,8,9].

In DPN, ROS production in the peripheral nerves is induced by multiple mechanisms [3,4]. Intrinsic factors, such as excess glucose and high fatty-acid flux, can markedly increase ROS and disrupt oxidative phosphorylation in the mitochondria. This process is hypothesized to be mediated by many complex mechanisms, including increased flux through the polyol pathway and the hexosamine biosynthetic pathway, protein kinase C (PKC) activation, and increased production of advanced glycation end products (AGEs). Furthermore, OS can activate multiple downstream kinases to elicit an unrestrained inflammatory reaction resulting in injury to the cells of the peripheral nerves [10]. In addition, antioxidant mechanisms are attenuated in DPN, therefore, compounding the issue of oxidative injury [11].

Small nerve fibers consist of myelinated Aδ fibers and unmyelinated C fibers. C fibers are responsible for sensing thermal nociception and cognizant pain after thermal or mechanical stimulation to the skin [12]. Dysfunctional small nerve fibers may manifest as small fiber neuropathy (SFN). In addition to invasive methods, such as skin biopsy, SFN may be evaluated quantitatively by noninvasive methods [13,14], including the use of a new electrode for intraepidermal electrical stimulation (IES) [15,16,17]. Nonmyelinated small nerve fibers in the distal foot are often first to be affected in DPN [18]. Consequently, increased pain threshold evoked by IES (PINT) was evident in both DPN and prediabetic subjects [17,19,20,21].

Without apparent disturbance to glucose metabolism, normal-high HbA1c levels in normoglycemic subjects can be a risk factor for increased PINT [17]. This indicates that the initial manifestation of DPN may occur in a normoglycemic state characterized by normal-high HbA1c levels rather than in a prediabetic state. We previously reported that the pathological factors for DPN were correlated differently with the elevation of PINT scores, depending on the stage of diabetes [21]. It remains unknown, however, whether OS or inflammation can be involved in elevated PINT scores in normoglycemic subjects similarly to in IFG subjects. It is of paramount importance to know such differences, because this may provide useful clues to intervene in normoglycemic subjects with elevated PINT scores.

In this study, we evaluated the correlation of elevated PINT scores with clinico-hematological factors relating to DPN, including OS and inflammation, in a rural Japanese population. We also explored factors responsible for eliciting OS in the same population.

## 2. Results

### 2.1. Clinical Profiles of the Study Participants

**Figure 1:**
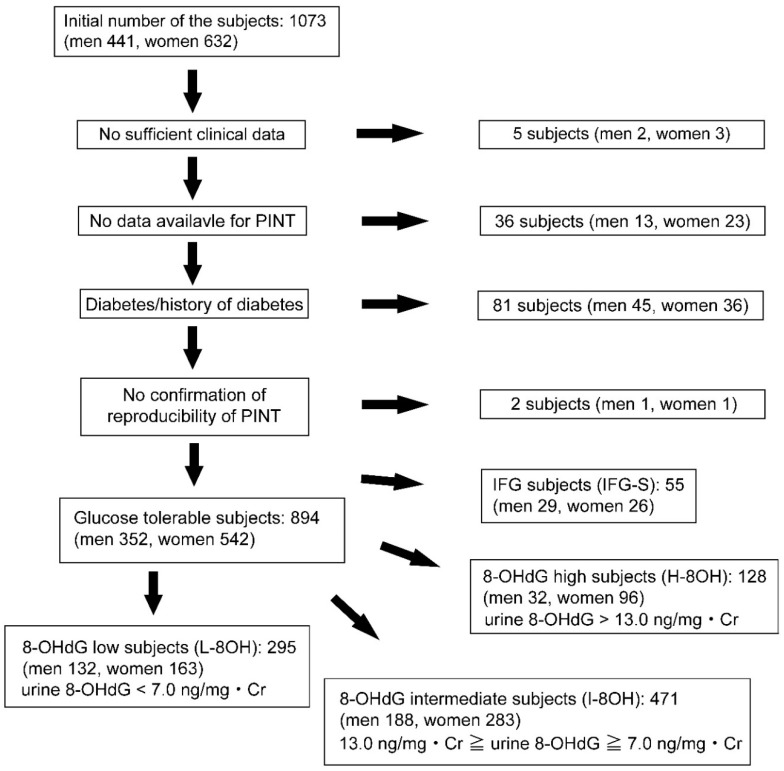
Examined subject selection.

The profile and selection of the subjects were described in our previous reports [17]. In brief, 894 normoglycemic subjects (352 men, 542 women) aged 53.8 ± 0.5 years were evaluated among 1073 volunteers (*n* = 1073) of the 2017 Iwaki study. Volunteers were excluded in this study based on the 2010 Japan Diabetes Society criteria (IFG: fasting blood glucose levels 110–125 mg/dL; diabetes: fasting blood glucose levels ≥126 mg/dL or HbA1c levels ≥6.5%) [22] (Figure 1). 

Eight hundred ninety-four normoglycemic subjects (352 men, 542 women) were finally examined out of 1073 volunteers from the Iwaki study 2017 in this study. The participants were further divided into urine 8-OHdG low subjects (L-8OH), urine 8-OHdG intermediate subjects (I-8OH), urine 8-OHdG high subjects (H-8OH), based on urine 8-OHdG levels. 55 IFG subjects (IFG-S) were used for the comparison to normoglycemic subjects. PINT, pain threshold from intraepidermal electrical stimulation: 8-OHdG, 8-hydroxy-2′-deoxyguanosine: IFG, impaired fasting glucose: Cr, creatinine.

We also excluded two subjects with fasting blood glucose levels lower than 63 mg/dL. After these exclusions, 55 subjects (29 men and 26 women) with IFG (IFG-S) aged 66.1 ± 1.4 years (*p* < 0.001 vs. normoglycemic subjects) were separately examined for the comparison of PINT scores with normoglycemic subjects. Normoglycemic subjects were further divided into three groups based on urine 8-OHdG levels as follows: (1) L-8OH (*n* = 295): urine 8-OHdG < 7.0 ng/mg·Cr, (2) I-8OH (*n* = 471): 13.0 ng/mg·Cr ≥ urine 8-OHdG ≥ 7.0 ng/mg·Cr, and (3) H-8OH (*n* = 128): urine 8-OHdG > 13.0 ng/mg·Cr.

Clinical profiles of men and women participants are shown in Table 1. The mean age was 51.9 ± 0.8 years for men and 53.7 ± 0.7 years for women. Waist circumference was higher in men compared to women (88.1 ± 0.5 vs. 81.0 ± 0.4 cm). FBG and sBP were higher in men than women, while pentosidine and urine 8-OHdG levels were lower in men (FBG: 92.4 ± 0.4 vs. 89.3 ± 0.4 mg/dL; sBP: 124.1 ± 0.9 vs. 119.4 ± 0.7 mmHg; pentosidine: 27.9 ± 0.6 vs. 30.6 ± 0.8 pmol/mL; urine 8-OHdG: 8.5 ± 0.2 vs. 9.3 ± 0.2 ng/mg Cr). The frequency of subjective symptoms and decreased ATR were comparable between men and women. PINT scores were also comparable between men and women.

The mean age was 51.9 ± 0.8 years for men and 53.7 ± 0.7 years for women. Waist circumference was higher in men compared to women (88.1 ± 0.5 vs. 81.0 ± 0.4 cm). FBG and sBP were higher in men than women, while pentosidine and urine 8-OHdG levels were lower in men (FBG: 92.4 ± 0.4 vs. 89.3 ± 0.4 mg/dL; sBP: 124.1 ± 0.9 vs. 119.4 ± 0.7 mmHg; pentosidine: 27.9 ± 0.6 vs. 30.6 ± 0.8 pmol/mL; urine 8-OHdG: 8.5 ± 0.2 vs. 9.3 ± 0.2 ng/mg Cr). The frequency of subjective symptoms and decreased ATR were comparable between men and women. PINT scores were also comparable between men and women.

### 2.2. Correlation of PINT Scores with Urine 8-OHdG Levels

Univariate regression analysis revealed a significant correlation between PINT scores and clinical measures, including age, BMI, waist circumference, FBG, HbA1c, sBP, urine 8-OHdG, presence of hypertension, and decreased ATR (Table 2). 

The correlation between PINT scores and HbA1c remained significant after adjustment for age and sex (β = 0.10, *p* = 0.01) (Table 3). 

Correlation between PINT scores and urine 8-OHdG levels also remained significant after adjustment for multiple factors (age, BMI, waist circumference, sBP, hypertension, FBG, and HbA1c; β = 0.09, *p* = 0.01) (Table 2 and Table 3).

### 2.3. Risk of Elevated PINT Scores as Indicated by Urine 8-OHdG Levels

**Figure 2:**
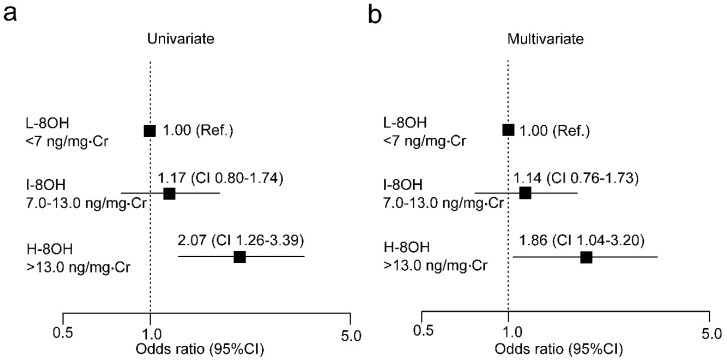
Logistic analysis of urine 8-OHdG and risk for increased PINT scores.Odds ratio with 95% confidence interval (CI) for urine 8-OHdG (**a**,**b**) are shown. Multiple factors are age, BMI, waist circumference, HbA1c, FBG, sBP, and hypertension. 8-OHdG, 8-hydroxydeoxy-2′-guanosine; Ref, reference; L-8OH: 8-OHdG low; I-8OH, 8-OHdG intermediate; H-8OH, 8-OHdG high; Cr, creatinine.

To explore the implication of urine 8-OHdG in altered PINT scores, we divided normoglycemic control participants into three groups (L-8OH < 7 ng/mg·Cr, I-8OH 7.0–13.0 ng/mg·Cr and H-8OH > 13.0 ng/mg·Cr). We conducted logistic regression analysis on graded levels of urine 8-OHdG for the risk of increased PINT scores (Figure 2). 

When high PINT scores were defined as 0.20 mA or greater, high urine 8-OHdG levels were a significant risk for elevated PINT threshold (Figure 2a). The risk remained significant after adjustment for multiple factors (age, BMI, waist circumference, HbA1c, FBG, sBP, and hypertension) (Figure 2b).

### 2.4. Correlation of Subjective Symptoms and Decreased ATR with Urine 8-OHdG Levels

The frequency of subjective neuropathic symptoms was comparable among stratified 8-OHdG groups (Table 4).

IFG subjects had a significantly higher frequency of subjective symptoms compared to either L-8OH or I-8OH subjects (*p* < 0.05, respectively). Additionally, decreased ATR in H-8OH subjects was significantly higher compared to L-8OH subjects (*p* < 0.05) (Table 5).

Decreased ATR of IFG subjects was significantly higher compared to both that of L-8OH subjects and I-8OH subjects (*p* < 0.01 and *p* < 0.05, respectively).

### 2.5. Clinical Factors Correlating with Urine 8-OHdG Levels

We explored clinical factors correlating with high urine 8-OHdG levels (Table 6).

Univariate regression analysis revealed a significant correlation between the levels of 8-OHdG in urine and clinical measures, such as gender, age, height, body weight, fat content, waist circumference, FBG, HbA1c, Tc, adiponectin, vitamin E, presence of hypertension, and dyslipidemia. The correlations between 8-OHdG and age, body weight, waist circumference and HbA1c remained significant after adjustment for multiple factors (gender, age, height, body weight, fat, waist circumference, FBG, HbA1c, Tc, sBP, adiponectin, vitamin E, hypertension, and dyslipidemia) (β = 0.25, *p* < 0.01: β = −0.36, *p* = 0.01: β = 0.31, *p* < 0.01: β = −0.08, *p* < 0.04, respectively).

### 2.6. Comparison of PINT Scores and Pathogenic Factors Implicated in DPN Development among Stratified Groups of Graded Urine 8-OHdG Levels and Presence of IFG

**Figure 3:**
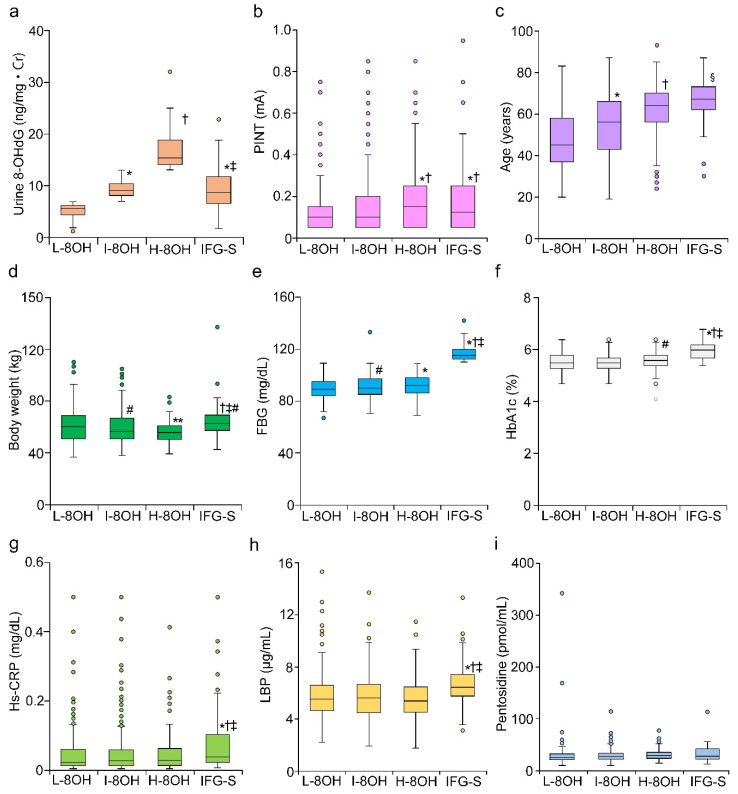
Comparison between IFG and each group of graded urine 8-OHdG levels in normoglycemic subjects. Normoglycemic subjects graded into three tertiles based on urine 8-OHdG levels and IFG state were compared regarding the factors correlating with high urine 8-OHdG and the mechanism of DPN. Urine 8-OHdG (**a**), PINT scores (**b**), age (**c**), body weight (**d**), FBG (**e**), HbA1c (**f**), Hs-CRP (**g**), LBP (**h**) and pentosidine (**i**) were evaluated. 8-OHdG, 8-hydroxy-2′-deoxyguanosine; L-8OH, 8-OHdG low; I-8OH, 8-OHdG intermediate; H-8OH, 8-OHdG high; IFG, impaired fasting glucose; Cr, creatinine; FBG, fasting blood glucose; Hs-CRP, high sensitive C-reactive protein; LBP, lipopolysaccharide-binding protein. Mean ± SEM, * *p* < 0.01 vs. L-8OH, ^†^ *p* < 0.01 vs. I-8OH, ^‡^ *p* < 0.01 vs. H-8OH, ^§^ *p* < 0.05 vs. H-8OH, ^#^ *p* < 0.05 vs. L-8OH, ** *p* < 0.05 vs. I-8OH.

Three groups based on graded urine 8-OHdG levels were examined to explore their relationship with PINT scores and known pathogenic factors that contribute to the onset and development of DPN (Figure 3).

The IFG group was also included for the comparison. Among our stratified groups, the H-8OH group had the highest 8-OHdG levels among the three groups (*p* < 0.01, L-8OH vs. I-8OH, and *p* < 0.01, H-8OH vs. I-8OH). Urine 8-OHdG levels in IFG subjects was similar to the level of I-8OH subjects, but was significantly lower compared to the level of H-8OH subjects (*p* < 0.01) (Figure 3a). Among the three groups, PINT scores were the highest in H-8OH subjects (*p* < 0.01 vs. L-8OH and I-8OH). PINT scores of IFG subjects were similar to that of H-8OH subjects (Figure 3b). Increased age of normoglycemic subjects correlated with increased urine 8-OHdG levels (*p* < 0.01 L-8OH vs. I-8OH and *p* < 0.01 H-8OH vs. I-8OH) (Figure 3c). The average age of IFG subjects was the highest among all groups (*p* < 0.05 vs. H-8OH). In contrast, body weight was inversely related to urine 8-OHdG levels, and H-8OH subjects had the lowest body weight among the three groups (*p* < 0.05 I-8OH vs. L-8OH and *p* < 0.05 H-8OH vs. I-8OH). IFG subjects were the heaviest among all groups (*p* < 0.05 vs. L-8OH) (Figure 3d). FBG was linearly related to the 8-OHdG levels (*p* < 0.01 L-8OH vs. H-8OH and *p* < 0.05 I-8OH vs. H-8OH). HbA1c was mildly increased as 8-OHdG levels increased (*p* < 0.05 L-8OH vs. H-8OH). (Figure 3e,f). FBG and HbA1c were significantly increased in IFG subjects compared to H-8OH subjects (*p* < 0.01) (Figure 3e,f). Hs-CRP, LBP, and pentosidine were all comparable among stratified 8-OHdG groups (Figure 3g–i). In contrast, Hs-CRP and LBP were significantly increased in IFG subjects compared to all normoglycemic groups (*p* < 0.01). No apparent increase in pentosidine in the IFG group was observed.

## 3. Discussion

In this study, we discovered that the PINT scores were significantly increased in normoglycemic subjects with high urine 8-OHdG levels to the same extent, as seen in IFG subjects. FBS and HbA1c were significantly increased in the H-8OH group; however, Hs-CRP, pentosidine, and LBP were not increased in the H-8OH group. The stratified logistic analysis further revealed correlations between elevated PINT scores and higher urine 8-OHdG levels. Urine 8-OHdG significantly correlated with the parameters of metabolic syndrome and impaired blood glucose metabolism.

In nondiabetic subjects, SFN can be manifested in amyloidosis, autoimmune diseases, HIV infection, paraneoplasia, Fabry disease, medications such as metronidazole and anti-cancer agents, alcohol, and vitamin deficiency [23]. In some of such pathological conditions, OS plays a pivotal role in the manifestation of SFN [24]. Our study first determined urine 8-OHdG levels were significantly correlated with SFN in nondiabetic participants. It is difficult, however, to ascribe the cause of the correlation between high urine 8-OHdG levels and increased PINT scores to such pathological conditions, because the participants in our study are healthy volunteers. In patients with type 2 diabetes, urine 8-OHdG was significantly increased compared to nondiabetic subjects and was proportional to HbA1c levels [25]. In our study, urine 8-OHdG levels significantly correlated with HbA1c level and FBG. Therefore, it is possible that OS may be exacerbated even by trivial disturbances in glucose metabolism in normoglycemic subjects.

Alternatively, urine 8-OHdG levels in the H-8OH group were significantly higher than that in the IFG group, despite the H-8OH group having lower HbA1c and FBG levels than the IFG group. These results suggest that factors other than impaired glucose metabolism may be implicated in the generation of OS in a subset of our population. Our multivariate linear regression analysis revealed significant associations between elevated urine 8-OHdG levels and body weight, waist circumference, the levels of HbA1c and Tc, and age in a sample of the rural Japanese population. Aging is a major risk factor for increased OS and development of DPN [26,27,28]. Nevertheless, the average age of IFG subjects was significantly higher than the average age of H-8OH subjects. Obesity is also correlated with urine 8-OHdG levels [29,30]. Cejvanovic et al. reported that BMI > 30 could be a risk for high urine 8-OHdG levels. In contrast, body weight was inversely related to urine 8-OHdG levels in our study. One explanation for this discrepancy is that only 3.9% (35 participants) of our population had BMI > 30. Therefore, demonstrating a correlation between urine 8-OHdG levels and obesity may be difficult in our study. Tg had an independently significant and positive correlation with urine 8-OHdG in asymptomatic Japanese subjects [31], while only Tc, but not LDL-c, HDL-c, and TG, was significantly correlated with urine 8-OHdG levels in our study. Collectively, these suggest that exploring factors which were not evaluated in the 2017 Iwaki study are needed in the future study.

Previous reports have shown that urine 8-OHdG is higher in diabetic patients with complications compared to those without complications [32,33,34]. Furthermore, OS is an important factor in the pathogenesis of DPN in conjunction with impaired nerve conduction and pathologic changes [4,6,7,8]. In humans, serum and urine 8-OHdG levels were significantly higher in prediabetic subjects with neuropathy compared to those without neuropathy [35]. In our study, the frequency of subjects with decreased ATR was significantly higher in the H-8OH group. 8-OHdG is released upon DNA repair or degradation and is excreted into urine [36]. Therefore, levels of 8-OHdG can reflect oxidative stress-induced tissue damage. This evidence may indicate that urine 8-OHdG levels can partially reflect the local state of OS in the peripheral nerves. To understand the precise role of OS in elevated pain threshold in normoglycemic subjects in future studies, a detailed evaluation of the peripheral nerve tissues will be required.

The polyol pathway, AGEs, PKC activation, inflammation, and OS all play an important role in DPN progression [3,4]. The contribution of those factors can be variable depending upon the stage of diabetes and the ethnicity of patients with DPN. The level of urine 8-OHdG in IFG subjects was lower than in H-8OH subjects despite high FBS and HbA1c level and similar elevation of PINT scores. On the other hand, inflammatory biomarkers, such as Hs-CRP and LBP, were increased in IFG subjects. These results suggest that OS plays a primary role in inducing PINT elevation in normoglycemic subjects, while both inflammation and OS cooperatively contribute to PINT elevation in IFG subjects. We previously showed the possibility that the more pathological factors for DPN could be gradually involved in proportion to the development of DPN [21,37]. Thus, early intervention may provide more benefit for treating SFN even in nondiabetic subjects, due to the simpler pathogenesis compared to the pathogenesis underlying PINT elevation in IFG or overtly diabetic patients.

We also evaluated antioxidant molecules in the blood of normoglycemic subjects. Ziegler et al. reported defects in antioxidant defense systems in DPN patients [11]. However, our previous study demonstrated there was no correlation between elevated PINT scores and the level of antioxidant molecules in diabetic patients [21]. In this study, antioxidant defense systems were intact in the normoglycemic subjects with abnormal urine 8-OHdG levels. Thus, the exaggerated generation of ROS is more important than a deficit in the antioxidant defense system in normoglycemic subjects with elevated PINT scores.

There are several limitations to this study. First, the study is a cross-sectional and single population study. To confirm the underlying role of OS in elevated PINT scores, we would need to follow the progression or reversal of altered PINT scores after lifestyle interventions in a detailed, longitudinal study. Second, the normoglycemic subjects in this study may incidentally include diabetic patients because diabetes was diagnosed only by a single measurement of FBG, HbA1c level, and clinical history. In future projects, PINT scores should be evaluated with an oral glucose tolerance test. Third, the specify of the PINT for SFN is not validated in this study. Previous studies showed that Aδ and C fibers were activated with IES, which evoked pain sensation [15,16]. However, our previous work showed that PINT was also correlated with decreased ATR, which could manifest in the pathological conditions other than SFN [17,21]. We need to confirm the presence of SFN with the pathological evaluation like intraepidermal nerve fiver density by skin biopsy or corneal confocal fluorescent microscopy in the future. These assessments may shed light on the pathological differences of SFN between normoglycemic and IFG subjects. Finally, we were relegated to evaluating the correlation of PINT scores with biomarkers from blood and urine because invasive procedures were not permitted in the Iwaki study. As previously mentioned, identifying the precise pathogenic mechanism underlying elevated PINT scores in normoglycemic subjects would require the evaluation of peripheral nerve tissues.

In conclusion, in normoglycemic study participants with high urine 8-OHdG levels, there was a significant correlation with elevated PINT scores, which was the similar degree to PINT scores observed in IFG subjects. The logistic analysis revealed that OS might be a risk factor and primary contributor to elevated PINT scores in normoglycemic subjects, and this may correlate with impaired glucose metabolism. The results reported herein suggest that interventions may be necessary for patients with high urine 8-OHdG levels, even if the disturbance of glucose metabolism is trivial.

## 4. Material and Methods

### 4.1. Demographic Characteristics of Study Participants

We recruited fasting normoglycemic and IFG subjects without a history of diabetes who previously participated as volunteers in the Iwaki study, a health promotion study of Japanese citizens over 10 years of age. In this project, health evaluation was conducted annually for the participants living in the Iwaki area, a suburban area of Hirosaki in the Aomori Prefecture of northern Japan [38]. Associations between clinical measurements and PINT scores were examined using the data of the 2017 Iwaki study. All subjects gave their informed consent for inclusion before they participated in the study. The study was conducted in accordance with the Declaration of Helsinki, and the protocol was approved by the Ethics Committee of the Hirosaki University School of Medicine (No. 2017-026).

### 4.2. Clinical Profile

Fasting blood samples were collected in the morning from peripheral veins in a supine position. The following clinical measures were recorded: Height, body weight, body mass index, waist circumference, percent body fat (fat), fasting blood glucose (FBG), Achilles tendon reflex, fasting serum insulin levels (F-IRI), HbA1c, systolic blood pressure (sBP), diastolic BP (dBP), serum levels of total cholesterol (Tc), triglyceride (Tg) levels, high-density lipoprotein cholesterol (HDL-c), low-density lipoprotein cholesterol (LDL-c), interleukin-6 (IL-6), high sensitivity C-reactive protein (Hs-CRP), adiponectin, lipopolysaccharide (LPS)-binding protein (LBP), vitamins A, C, and E, lutein, zeaxanthin, β-cryptoxanthin, α-carotene, β-carotene, and lycopene, and urine levels of 8-OHdG. Adipose tissue volume was measured by the bioelectricity impedance method using a Tanita MC-190 body composition analyzer (Tanita Corp., Tokyo, Japan). Achilles tendon reflex (ATR) was scored based on two titer systems: (1) score 0: areflexia/hyporiflexia; and (2) score 1: normal/hyperreflexia. HbA1c (%) was expressed as the National Glycohemoglobin Standardization Program value. Indices of insulin resistance and secretion were assessed by the homeostasis model assessment using FBG and insulin levels (HOMA-IR and HOMA-β), respectively. According to a previous report, we defined high urine 8-OHdG values as more than 13.0 ng/mg·Cr [39]. Because there was no definition of low urine 8-OHdG levels in previous studies, we defined low 8-OHdG values as less than 7.0 ng/mg·Cr, which enabled to significantly differentiate the participants with low 8-OHdG levels from others. Consequently, about 50% of the total participants were ranked as intermediate 8-OHdG levels. None of the diagnosed with type 1 diabetes or inherited diseases that affected HbA1c values. Hypertension was defined as blood pressure ≥140/90 mmHg or a history of treatment for hypertension. Hyperlipidemia was defined as Tc ≥ 220 mg/dL, Tg ≥ 150 mg/dL, or history of treatment for hyperlipidemia. Alcohol intake (current or nondrinker), smoking habits (current or nonsmoker), and subjective neuropathic symptoms were determined via questionnaire.

### 4.3. PINT Measurement

For nociceptive stimulation, the IES method was adopted using a disposable concentric bipolar needle electrode (NM-983W; Nihon Kohden Corp., Tokyo, Japan) connected to a specific stimulator for cutaneous Aδ and C fibers as previously described (PNS-7000; Nihon Kohden) [15,16,17,19,20,21]. The stimulator consisted of an outer ring anode (1.3 mm diameter) and the cathode of an inner needle that protruded 0.025 mm from the level of the outer ring. The IES electrode was placed onto the skin of the instep (over the extensor digitorum brevis) to deliver weak continuous electrical stimulations. This stimulation can evoke a local pricking sensation. In instances where the keratinized layer of the skin was too thick and likely to interrupt the electronic stimulation, the electrode was moved elsewhere on the same foot to locate a thinner area. The participants were instructed to push the button as quickly as possible only when they felt a sensation. Stimulation intensity was decreased by 0.05 mA stepwise from 0.4 mA until the participants reported a pricking sensation. The current intensity is directly proportional to the intensity of stimulation. PINT scores were defined as the minimum intensity at which the participants felt a pricking sensation in more than two trials. Therefore, PINT can evaluate the degree of hypoalgesia in response to electrical pain stimulation. A total of 20 well-trained staff were involved in the measurement of PINT.

### 4.4. Statistical Methods

The values of clinical measures are expressed as means ± SEM. The statistical significance of the difference in values between two groups (parametric) and case-control associations among groups (nonparametric) was assessed by nonparametric t-tests and one way analysis of variance (ANOVA) with post hoc tests and χ^2^ tests, respectively. Correlations between PINT scores and clinical parameters, and urine 8-OHdG levels and clinical parameters were assessed by linear regression analyses, which were further calculated by multiple logistic regression analysis. Values were adjusted for factors identified to associate with PINT scores and urine 8-OHdG levels using univariate regression analysis and accounting for potentially cofounding variables for SFN, as reported in a previous study [17]. For statistical analyses, urine 8-OHdG was log-transformed (log10) to approximate a normal distribution. The risk of higher urine 8-OHdG and increased PINT scores were calculated by multiple logistic regression analysis with adjustment for factors found to be associated with PINT scores using univariate regression analysis. To calculate the odds ratio, elevated PINT scores were designated as 0.20 mA or greater, which was determined from our previous study [17]. A value of *p* < 0.05 was regarded as statistically significant. All analyses were done using JMP version 10.0.4 and StatView version 5.0.1 (SAS Institute Inc., Cary, NC, USA).

## Figures and Tables

**Table 1:** Clinical profiles of examined subjects.

	Men	Women	*p*
Numbers	352	542	-
Age (yrs)	51.86 ± 0.81	53.71 ± 0.65	0.075
Height (cm)	169.08 ± 0.36	156.12 ± 0.27	<0.0001
Body weight (kg)	67.90 ± 0.58	53.63 ± 0.36	<0.0001
BMI (kg/m^2^)	23.73 ± 0.18	22.03 ± 0.15	<0.0001
Fat (%)	23.73 ± 0.18	22.03 ± 0.15	<0.0001
Waist circumference (cm)	88.11 ± 0.49	80.98 ± 0.40	<0.0001
FBG (mg/dL)	92.40 ± 0.43	89.29 ± 0.35	<0.0001
HbA1c (%)	5.53 ± 0.02	5.56 ± 0.01	0.2082
F-IRI (μU/mL)	4.88 ± 0.17	4.92 ± 0.09	0.8405
HOMA-β	61.88 ± 2.27	70.54 ± 0.14	0.0006
HOMA-IR	1.13 ± 0.04	1.10 ± 0.02	0.5657
sBP (mmHg)	124.13 ± 0.93	119.42 ± 0.73	<0.0001
dBP (mmHg)	74.42 ± 0.63	69.23 ± 0.46	<0.0001
Tc (mg/dL)	203.42 ± 1.78	209.35 ± 1.48	0.0111
Tg (mg/dL)	121.06 ± 4.79	78.94 ± 1.83	<0.0001
HDL-c (mg/dL)	59.95 ± 0.89	70.83 ± 0.70	<0.0001
LDL-c (mg/dL)	114.05 ± 1.52	116.05 ± 1.23	0.3068
IL-6 (pg/mL)	1.64 ± 0.21	1.40 ± 0.20	0.4156
Hs-CRP (mg/dL)	0.06 ± 0.01	0.05 ± 0.01	0.0762
Adiponectin (μg/mL)	8.58 ± 0.02	13.63 ± 0.28	<0.0001
Pentosidine (pmol/mL)	27.92 ± 0.58	30.60 ± 0.77	0.0120
Urine 8-OHdG (ng/mg·Cr)	8.48 ± 0.18	9.33 ± 0.19	0.0019
Vitamin A (μg/mL)	0.52 ± 0.01	0.39 ± 0.01	<0.0001
Vitamin C (μg/mL)	10.10 ± 0.20	12.88 ± 0.19	<0.0001
Vitamin E (μg/mL)	11.79 ± 0.19	11.73 ± 0.14	0.7934
Lutein (μg/mL)	0.24 ± 0.01	0.28 ± 0.01	<0.0001
Zeaxantihin (μg/mL)	0.05 ± 0.01	0.05 ± 0.01	0.6634
β-Cryptoxanthin (µg/mL)	0.11 ± 0.01	0.16 ± 0.01	<0.0001
α-Carotene (µg/mL)	0.12 ± 0.01	0.16 ± 0.01	<0.0001
β-Carotene (µg/mL)	0.26 ± 0.01	0.50 ± 0.01	<0.0001
Lycopene (µg/mL)	0.22 ± 0.01	0.24 ± 0.01	0.006
LBP (µg/mL)	5.78 ± 0.08	5.59 ± 0.07	0.0961
Hypertension: n (%)	26.14	22.51	0.2454
Dyslipidemia: n (%)	8.86	10.07	0.6265
Alcohol habit: n (%)	69.14	32.97	<0.0001
Smoking habit: n (%)	37.14	23.08	<0.0001
Pack a year	13.66 ± 1.21	2.52 ± 0.28	<0.0001
Subjective symptoms: n (%)	1.42	1.85	0.8287
Decreased ATR: n (%)	21.59	14.94	0.2745
PINT	0.156 ± 0.01	0.14 ± 0.01	0.1635

BMI, body mass index; FBG, fasting plasma glucose; F-IRI, fasting serum insulin; HOMA-β, homeostatic model assessment β cell function; HOMA-IR, homeostatic model assessment insulin resistance; sBP, systolic blood pressure; dBP, diastolic blood pressure; Tc, total cholesterol; Tg, triglyceride; HDL-c, high-density lipoprotein cholesterol; LDL-c, low-density lipoprotein cholesterol; IL-6, interleukin-6; Hs-CRP, high sensitivity C-reactive protein; Urine 8-OHdG, urine 8-hydoroxy-2′-deoxyguanosine; LBP, lipoporysaccharide-binding protein; ATR, Achilles tendon reflex; PINT, pain threshold from intraepidermal electrical stimulation.

**Table 2:** Clinical factors correlated with PINT scores.

	Univariate		Multivariate	
Characteristics	β	*p*	β	*p*
Gender (male/female)	0.0488	0.1446	-	-
Age (years)	0.47610	0.001	0.040763	0.3576
Height (cm)	−0.02268	0.4978	-	-
Body weight (kg)	0.059134	0.0769	-	-
BMI (kg/m^2^)	0.07997	0.0168	−0.00724	0.9235
Fat (%)	0.030964	0.3554	-	-
Waist circumference (cm)	0.09508	0.0044	0.05574	0.4688
FBG (mg/dL)	0.09668	0.0038	0.01272	0.7469
HbA1c (%)	0.1202	0.0003	0.07549	0.0487
F-IRI (μU/mL)	0.026419	0.4294	-	-
HOMA-β	−0.02683	0.4228	-	-
HOMA-IR	0.039726	0.2346	-	-
sBP (mmHg)	0.091228	0.0063	0.03510	0.3667
dBP (mmHg)	0.03714	0.2665	-	-
Tc (mg/dL)	0.001451	0.9654	-	-
Tg (mg/dL)	0.044434	0.1836	-	-
HDL-c (mg/dL)	−0.0549	0.1004	-	-
LDL-c (mg/dL)	0.022807	0.4951	-	-
IL-6 (pg/mL)	0.029864	0.3717	-	-
Hs-CRP (mg/dL)	0.028409	0.3954	-	-
Adiponectin (μg/mL)	−0.02645	0.4288	-	-
Pentosidine (pmol/mL)	0.023021	0.4920	-	-
Urine 8-OHdG (ng/mg·Cr)	0.119537	0.0003	0.09352	0.0078
Vitamin A (μg/mL)	−0.00801	0.8111	-	-
Vitamin C (μg/mL)	−0.02627	0.4331	-	-
Vitamin E (μg/mL)	0.048907	0.1442	-	-
Lutein (μg/mL)	0.041578	0.2145	-	-
Zeaxantihin (μg/mL)	−0.00933	0.7806	-	-
β-Cryptoxanthin (µg/mL)	0.026938	0.4214	-	-
α-Carotene (µg/mL)	0.019454	0.5615	-	-
β-Carotene (µg/mL)	0.027926	0.4046	-	-
Lycopene (µg/mL)	−0.03013	0.3685	-	-
LBP (µg/mL)	0.0973	0.0559	-	-
Hypertension: n (%)	0.068749	0.0399	−0.01451	0.7105
Dyslipidemia: n (%)	0.023986	0.4731	-	-
Alcohol habit: n (%)	−0.01053	0.7528	-	-
Smoking habit: n (%)	0.295599	0.1128	-	-
Pack a year	−0.03481	0.2976	-	-
Subjective symptoms	0.019313	0.5641	-	-
Decreased ATR	−0.07009	0.0362	−0.02693	0.4338

BMI, body mass index; FBG, fasting plasma glucose; F-IRI, fasting serum insulin; HOMA-β, homeostatic model assessment β cell function; HOMA-IR, homeostatic model assessment insulin resistance; sBP, systolic blood pressure; dBP, diastolic blood pressure; Tc, total cholesterol; Tg, triglyceride; HDL-c, high-density lipoprotein cholesterol; LDL-c, low-density lipoprotein cholesterol; IL-6, interleukin-6; Hs-CRP, high sensitivity C-reactive protein; Urine 8-OHdG, urine 8-hydoroxy-2′-deoxyguanosine; LBP, lipoporysaccharide-binding protein and ATR, Achilles tendon reflex.

**Table 3:** Correlation of urine 8-OHdG with PINT scores.

	Univariate		Age and Gender Adjusted		Multivariate	
	β	*p*	β	*p*	β	*p*
Urine 8-OHdG (ng/mg·Cr)	0.119537	0.0003	0.0946	0.0068	0.0944	0.0071

8-OHdG, 8-hydroxy-2′-deoxyguanosine; PINT, pain threshold from intraepidermal electrical stimulation.

**Table 4:** Correlation of high urine 8-OHdG and IFG with subjective neuropathic symptoms.

	Subjective Symptoms		Total
	−	+	
Control	98.5% (881)	1.5% (13)	100% (894)
L-8OH	99.0% (292)	1.0% (3)	100% (295)
I-8OH	98.9% (466)	1.1% (5)	100% (471)
H-8OH	96.1% (123)	3.9% (5)	100% (128)
IFG-S	90.2% (46)	9.8% (5) *	100% (51)

* *p* < 0.05 vs. L-8OH and I-8OH. 8-OHdG, 8-hydroxydeoxy-2′-guanosine; IFG, impaired fasting glucose.

**Table 5:** Correlation of high urine 8-OHdG and IFG with decreased ATR.

	ATR		Total
	Normal	Weak	
Control	82.4% (737)	17.6% (157)	100% (894)
L-8OH	85.9% (255)	14.1% (42)	100% (297)
I-8OH	82.4% (388)	17.6% (83)	100% (471)
H-8OH	74.6% (94)	25.4% (32) *	100% (126)
IFG-S	64.7% (33)	35.3% (18) ^†^	100% (51)

* *p* < 0.05 vs. L-8OH, ^†^ *p* < 0.01 vs. L-8OH, *p* < 0.05 vs. I-8OH. 8-OHdG, 8-hydroxydeoxy-2′-guanosine; ATR, Achilles tendon reflex; IFG, impaired fasting glucose.

**Table 6:** Clinical factors correlated with urine 8-OHdG levels.

	Univariate		Multivariate	
Characteristics	β	*p*	β	*p*
Gender (male/female)	−0.09935	0.0029	−0.03067	0.6863
Age (years)	0.312015	<0.0001	0.253719	<0.0001
Height (cm)	−0.19183	<0.0001	0.104831	0.1370
Body weight (kg)	−0.09598	0.0041	−0.3565	0.0010
BMI (kg/m^2^)	0.029451	0.3791	-	-
Fat (%)	0.137257	<0.0001	0.049914	0.5104
Waist circumference (cm)	0.07414	0.0266	0.307457	0.0006
FBG (mg/dL)	0.114334	0.0006	0.046716	0.2183
HbA1c (%)	0.072723	0.0297	−0.07707	0.0402
F-IRI (μU/mL)	0.01646	0.6231	-	-
HOMA-β	−0.04929	0.1413	-	-
HOMA-IR	0.033675	0.3145	-	-
sBP (mmHg)	−0.03965	0.2362	-	-
dBP (mmHg)	0.012238	0.7168	-	-
Tc (mg/dL)	0.084499	0.0115	−0.04024	0.3066
Tg (mg/dL)	−0.0263	0.4322	-	-
HDL-c (mg/dL)	0.051609	0.1231	-	-
LDL-c (mg/dL)	0.059956	0.0732	-	-
IL-6 (pg/ml)	0.018127	0.5883	-	-
Hs-CRP (mg/dL)	−0.01736	0.6042	-	-
Adiponectin (g/mL)	0.125921	0.0002	0.05128	0.1858
Pentosidine (pmol/mL)	0.058815	0.0788	-	-
Vitamin A (μg/mL)	0.029435	0.3794	-	-
Vitamin C (μg/mL)	−0.0279	0.4048	-	-
Vitamin E (μg/mL)	0.115518	0.0005	0.044903	0.2436
Lutein (μg/mL)	0.062833	0.0604	-	-
Zeaxantihin (μg/mL)	0.012228	0.7150	-	-
β-Cryptoxanthin (µg/mL)	0.063845	0.0564	-	-
α-Carotene (µg/mL)	−0.05329	0.1114	-	-
β-Carotene (µg/mL)	0.047566	0.1553	-	-
Lycopene (µg/mL)	−0.06301	0.0597	-	-
LBP (µg/ml)	−0.02992	0.3724	-	-
Hypertension: n (%)	0.121086	0.0003	−0.04844	0.1962
Dyslipidemia: n (%)	0.081815	0.0144	0.000961	0.9772
Alcohol habit: n (%)	−0.0523	0.1181	-	-
Smoking habit: n (%)	0.013817	0.6803	-	-
Pack a year	0.037952	0.2570	-	-
Subjective symptoms	0.021403	0.5227	-	-
Decreased ATR	−0.10795	0.0012	-	-
PINT	0.10258	0.0021	-	-

BMI, body mass index; FBG, fasting plasma glucose; F-IRI, fasting serum insulin; HOMA-β, homeostatic model assessment β cell function; HOMA-IR, homeostatic model assessment insulin resistance; sBP, systolic blood pressure; dBP, diastolic blood pressure; Tc, total cholesterol; Tg, triglyceride; HDL-c, high-density lipoprotein cholesterol; LDL-c, low-density lipoprotein cholesterol; IL-6, interleukin-6; Hs-CRP, high sensitivity C-reactive protein; Urine 8-OHdG, urine 8-hydoroxy-2′-deoxyguanosine; LBP, lipoporysaccharide-binding protein; ATR, Achilles tendon reflex and PINT, pain threshold of intraepidermal electrical stimulation.

## Abbreviations

8-OHdG	8-hydroxy-2′-deoxyguanosine
AGEs	advanced glucose end products
ATR	Achilles tendon reflex
BMI	body mass index
dBP	diastolic BP
DPN	diabetic polyneuropathy
FBG	fasting blood glucose
F-IRI	fasting serum insulin levels
HbA1c	glycohemoglobin A1c
HDL-c	high-density lipoprotein-cholesterol
HOMA-β	homeostasis model assessment using insulin levels
HOMA-IR	homeostasis model assessment using fasting blood glucose
Hs-CRP	high-sensitivity C reactive protein
IES	electrode for intraepidermal electrical stimulation
IFG	impaired fasting glucose
IL-6	interleukin-6
LBP	LPS-binding protein
LDL-c	low-density lipoprotein-cholesterol
LPS	Lipopolysaccharide
PINT	pain threshold evoked by IES
PKC	protein kinase C
ROS	reactive oxygen species
sBP	systolic blood pressure
SFN	small fiber neuropathy
Tc	total cholesterol
Tg	triglyceride
